# Defect Passivation on Lead-Free CsSnI_3_ Perovskite Nanowires Enables High-Performance Photodetectors with Ultra-High Stability

**DOI:** 10.1007/s40820-022-00964-9

**Published:** 2022-11-07

**Authors:** Zheng Gao, Hai Zhou, Kailian Dong, Chen Wang, Jiayun Wei, Zhe Li, Jiashuai Li, Yongjie Liu, Jiang Zhao, Guojia Fang

**Affiliations:** 1 International School of Microelectronics, Dongguan University of Technology, Dongguan, 523808 Guangdong People’s Republic of China; 2 Key Lab of Artificial Micro- and Nano-Structures of Ministry of Education of China, School of Physics and Technology, Wuhan University, Wuhan, 430072 People’s Republic of China; 3 Faculty of Physics and Electronic Science, Hubei University, Wuhan, 430062 People’s Republic of China

**Keywords:** Pb-free, Perovskite, CsSnI_3_, Photodetector, Nanowire

## Abstract

In recent years, Pb-free CsSnI_3_ perovskite materials with excellent photoelectric properties as well as low toxicity are attracting much attention in photoelectric devices. However, deep level defects in CsSnI_3_, such as high density of tin vacancies, structural deformation of SnI_6_^−^ octahedra and oxidation of Sn^2+^ states, are the major challenge to achieve high-performance CsSnI_3_-based photoelectric devices with good stability. In this work, defect passivation method is adopted to solve the above issues, and the ultra-stable and high-performance CsSnI_3_ nanowires (NWs) photodetectors (PDs) are fabricated via incorporating 1-butyl-2,3-dimethylimidazolium chloride salt (BMIMCl) into perovskites. Through materials analysis and theoretical calculations, BMIM^+^ ions can effectively passivate the Sn-related defects and reduce the dark current of CsSnI_3_ NW PDs. To further reduce the dark current of the devices, the polymethyl methacrylate is introduced, and finally, the dual passivated CsSnI_3_ NWPDs show ultra-high performance with an ultra-low dark current of 2 × 10^–11^ A, a responsivity of up to 0.237 A W^−1^, a high detectivity of 1.18 × 10^12^ Jones and a linear dynamic range of 180 dB. Furthermore, the unpackaged devices exhibit ultra-high stability in device performance after 60 days of storage in air (25 °C, 50% humidity), with the device performance remaining above 90%.

**Supplementary Information:**

The online version contains supplementary material available at 10.1007/s40820-022-00964-9.

## Highlights

- Through materials analysis and theoretical calculations, the defects of CsSnI_3_ nanowires (NWs) were effectively passivated via incorporating 1-butyl-2,3-dimethylimidazolium chloride into perovskites.
- The high-performance CsSnI_3_ NW photodetectors (PDs) were achieved with a responsivity of up to 0.237 A W^−1^, a high detectivity of 1.18 × 10^12^ Jones and a linear dynamic range of 180 dB. These values are comparable to the reported high-performance Pb-based perovskite PDs and higher than those of the Pb-free perovskite PDs.
- Our unpackaged devices exhibit ultra-high stability with no degradation after 60 days of storage in air (25 °C, 50% humidity).

**Supplementary Information:**

The online version contains supplementary material available at 10.1007/s40820-022-00964-9.

## Graphical abstract

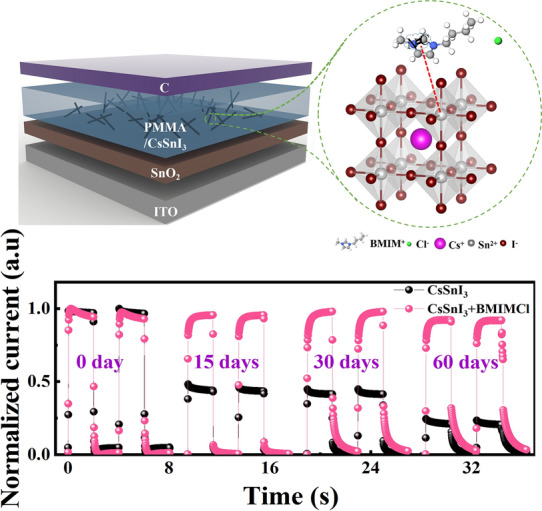

## Introduction

Because of excellent photoelectronic properties and low fabrication cost, organic–inorganic hybrid perovskites have become a hot topic in recent years and are widely used in solar cells [1–4], photodetectors (PDs) [5–7], light-emitting diodes (LEDs) [8, 9], etc. Until now, the highest certified power conversion efficiency of perovskite solar cells has exceeded 25% [10, 12], and the LEDs based on lead-containing perovskite materials also show high performance; for example, the highest external quantum efficiency of green LEDs has exceeded 20% [13–15]. However, the presence of heavy metal lead severely limits the commercialization of lead halide materials, which will face security risks of heavy metal lead leakage during mass production, transportation, installation and operation.

In this context, lead-free perovskite materials based on tin (Sn) [14], bismuth [15], germanium [16], antimony [17] or copper [18] have attracted much attention. Among these lead-free perovskite materials, CsSnI_3_ is more popular in photovoltaic applications due to its similar crystal and electronic structure to its Pb-based counterpart. Besides, inorganic perovskite CsSnI_3_ has a narrow optical band gap close to the Shockley–Queisser limit, with long lifetime and high charge carrier mobility [19, 20]. Moreover, the melting point of the CsSnI_3_ is up to 451 °C, which means that it has excellent inherent thermal stability. Therefore, the development of the inorganic perovskite CsSnI_3_ shows great prospect, and many scientists have achieved meaningful results [21–23]. Jin et al. [24] reported the growth of CsSnX_3_ (X = Br, I) perovskite semiconductors with controlled orientation and size by high-temperature vapor-phase epitaxy on mica sheets. Yang et al. [25] reported the preparation of CsSnX_3_ (X = Cl, Br, and I) perovskite nanowire (NW) arrays by chemical vapor deposition with a responsivity of 54 mA W^−1^, a detectivity of 3.85 × 10^–5^ Jones, and fast rise and decay time constants of 83.8 and 243.4 ms, respectively. However, the efficiency of the solution-processed CsSnI_3_ devices was much lower than that of the Pb-based analogs, mainly due to the weak Sn-I bond of inorganic CsSnI_3_, which causes lower tin vacancy formation energy and the easy oxidation of Sn^2+^ to Sn^4+^, leading to a high level of self-P doping in inorganic CsSnI_3_ perovskites, and the reduction in the device performance and the decrease in the output stability [26]. Therefore, strategies to passivate the Sn defects in inorganic CsSnI_3_ perovskites while maintaining their environmental stability are urgently needed.

In this work, 1-butyl-2,3-dimethylimidazolium chloride (BMIMCl) salt is introduced to passivate the defects of perovskite CsSnI_3_ NWs. Through materials analysis and theoretical calculations, the BMIMCl has a strong passivating effect on Sn-related defects via large π-bonds in N–C = N, and the lone pair of electrons in large π-bonds enhances the electron density around Sn^2+^ in CsSnI_3_ and protects it from oxidation to Sn^4+^; thus, the fabricated CsSnI_3_ NWs with BMIMCl show high light absorption, low defect density and air stability. To further reduce the dark current of the devices, the polymethyl methacrylate (PMMA) was applied, and finally, the dual passivated CsSnI_3_ NW PDs show ultra-high performance with an ultra-low dark current of 2 × 10^–11^ A, a high responsivity of 0.237 A W^−1^, a high detectivity of 1.18 × 10^12^ Jones and a linear dynamic range (LDR) of 180 dB. Besides, our unpackaged devices exhibit good stability with less than 10% degradation in device performance after 60 days of storage in air (25 °C, 50% humidity), demonstrating good application potential.

## Experimental and Calculation

### Device Fabrication

A pre-etched indium-tin oxide (ITO) glass substrate was ultrasonically cleaned with detergent, deionized water, ethanol and iso-propyl alcohol for 15 min, respectively. To prepare the SnO_2_ precursor solution, the SnO_2_ stock solution (1 mL) was diluted in deionized water (4 mL). The as-cleaned ITO substrate was treated with UV ozone at 100 °C for 10 min. A compact layer of SnO_2_ was spin-coated on top of the ITO at 4000 rpm for 30 s. Then, it was heated at 150 °C for 30 min in air. After that, the samples were treated with UV ozone for 10 min. Subsequently, the samples were transferred into a N_2_ filled glovebox with H_2_O and O_2_ concentrations of < 0.1 ppm. A layer of PbI_2_ film was fabricated by spin-coating PbI_2_/BMIMCl (1 mol mL^−1^/0, 5, 8, 10 and 15 mg mL^−1^) in DMF at 3000 rpm for 30 s, followed by annealing at 70 °C for 10 min. Then, the substrate was soaked in the prepared CsI/SnI_2_/SnF_2_ (5 /4 /0.4 mg mL^−1^) solution in anhydrous methanol for 2 h. After that, the substrates were placed in an isopropyl alcohol solution for 20 s and then annealed at 180 °C for 10 min. Finally, a layer of PMMA was coated on the samples by spin-coating PMMA in CB (20 mg mL^−1^) at 2000/3000/4000/5000/6000/7000 rpm for 30 s, followed by annealing at 100 °C for 10 min. Then, a layer of carbon electrode was scraped on the samples and annealed at 120 °C for 15 min in air.

### Calculations

All the calculations are performed in the framework of the density functional theory (DFT) with the projector augmented plane-wave method, as implemented in the Vienna ab initio simulation package. The generalized gradient approximation proposed by Perdew, Burke and Ernzerhof is selected for the exchange–correlation potential. The long-range van der Waals interaction is described by the DFT-D3 approach. The cutoff energy for plane wave is set to 400 eV. The energy criterion is set to 10^–5^ eV in iterative solution of the Kohn–Sham equation. The Brillouin zone integration is performed at the Gamma point for structural optimization, and a 3 × 3 × 1 k-mesh grid is used for electronic structure calculations. All the structures are relaxed until the residual forces on the atoms have declined to less than 0.05 eV Å^−1^.

## Results and Discussion

**Fig. 1:**
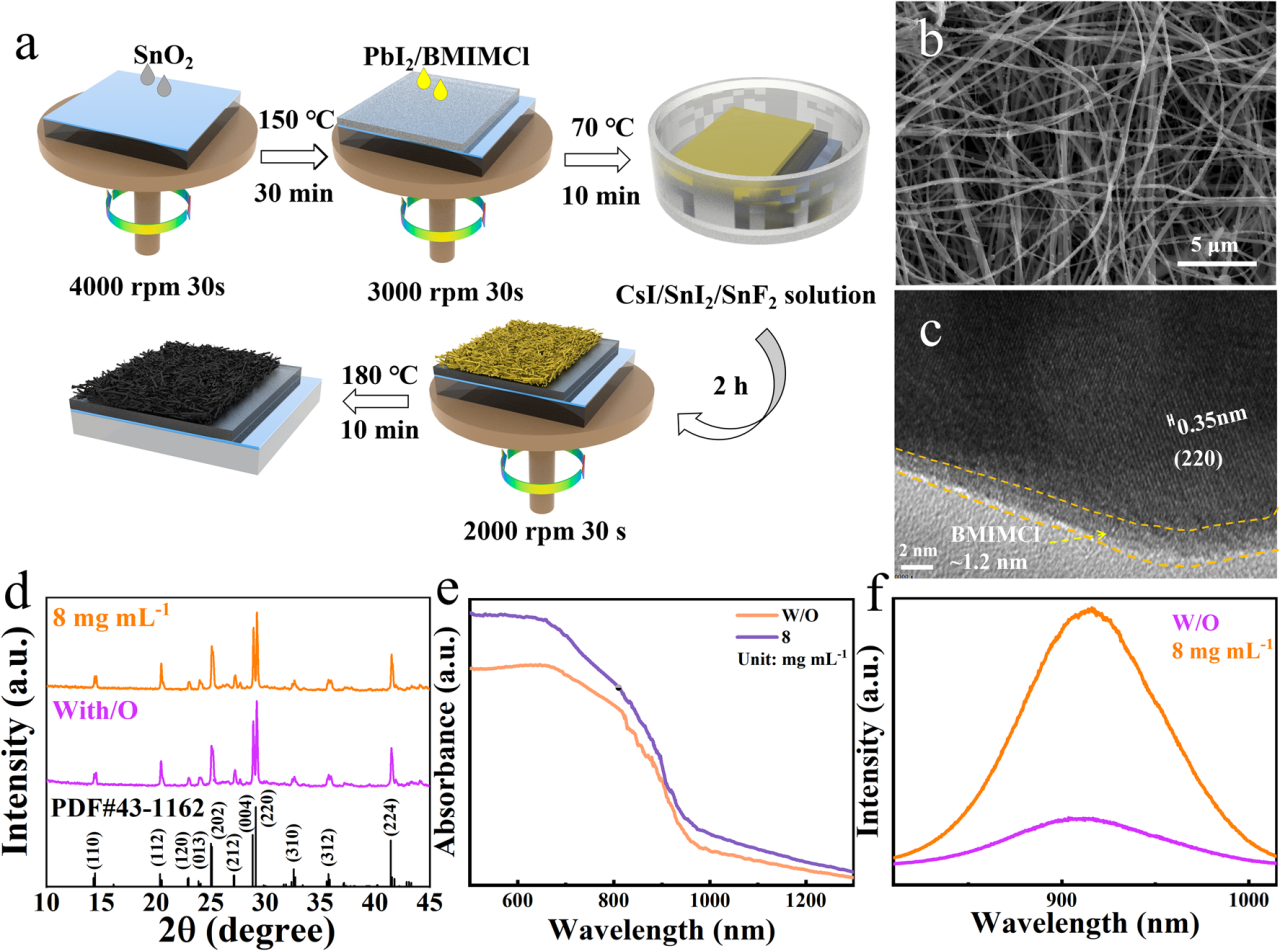
**a** Flow chart for the preparation of CsSnI_3_ NWs. **b** Surface SEM and **c** HRTEM of CsSnI_3_ NWs with BMIMCl. **d** XRD, **e** optical absorption and **f** steady-state PL curves of CsSnI_3_ NWs with or without 8 mg mL^−1^ BMIMCl

A schematic diagram of the preparation of CsSnI_3_ NWs by a two-step solution method is shown in Fig. 1a. The aqueous SnO_2_ solution was first spin-coated onto a clean ITO substrate and then annealed on a hot table at 150 °C for 30 min to obtain the SnO_2_ films. The PbI_2_/BMIMCl (1 mol mL^−1^/0, 5, 8, 10 and 15 mg mL^−1^) DMF solution was spin-coated on the SnO_2_/ITO substrate and then annealed at 70 °C for 10 min to obtain the PbI_2_-BMIMCl films. The PbI_2_-BMIMCl film-covered substrates were immersed in a methanol solution of CsI/SnI_2_/SnF_2_ (5/4/0.4 mg mL^−1^) for 2 h. The yellow phase CsSnI_3_ (γ-CsSnI_3_) was obtained by the B-position exchange reaction. During the reaction, the perovskite also grows in a specific direction because of the strong anisotropy of γ-CsSnI_3_ and eventually forms one-dimensional γ-CsSnI_3_ NWs [27, 28]. After that, the CsSnI_3_ NWs-covered substrates were placed in an isopropyl alcohol solution for 20 s to wash away the impurities on it and then annealed at 180 °C for 10 min to obtain the black phase CsSnI_3_ NWs (B-CsSnI_3_ NWs). The top-view scanning electron microscopy (SEM) images of CsSnI_3_ NW films with different soaking times (2, 4, 8, 16, 24, and 48 h) are shown in Figs. 1b and S1, which show little changes in surface morphology. Besides, the energy-dispersive spectroscopy (EDS) results (Table S1) show that when the soaking time is above 2 h, the perovskite NWs indicate very little Pb content (~ 0.21%) and we think our perovskite nanowires are basically all converted to CsSnI_3_ NWs and 2 h of soaking time are enough.

When different BMIMCl concentrations (5, 8, 10 and 15 mg mL^−1^) are incorporated (Fig. S2), the surface coverage of the nanowires is more obvious with the increase in the concentration. In order to further study the effect of the incorporation of BMIMCl, the high-resolution transmission electron microscopy (HRTEM) is performed, and the results are shown in Figs. 1c and S4. From these figures, all perovskite NW samples show high crystallinity. The d-spacing value of the CsSnI_3_ without BMIMCl is estimated to be 0.31 nm, corresponding to the (202) plane of CsSnI_3_. For the BMIMCl + CsSnI_3_ sample, the spacing between the lattice fringe is 0.35 nm, which corresponds to the (220) plane of CsSnI_3_, and shows clearer lattice fringes of the perovskite grains, indicating higher crystallinity of the perovskite grains [29, 30]. Besides, the NW is covered by an amorphous BMIMCl molecular layer with a thickness of about 1.2 nm. This thin layer of BMIMCl will protects the underlying CsSnI_3_ from degradation when exposed to ambient air.

Figures 1d and S3a show the X-ray diffraction (XRD) patterns of CsSnI_3_ NW films with different concentrations of BMIMCl, which match well with the orthorhombic perovskite crystal structure. After the addition of BMIMCl, the peak intensity of the (220) plane of the CsSnI_3_ NW film is slightly enhanced compared with that of the unincorporated CsSnI_3_ NW film, the better crystal quality of the perovskite may improve the carrier transmission of the CsSnI_3_ [31]. Figures 1e and S3b show the optical properties of the perovskite with different concentrations of BMIMCl, which show the CsSnI_3_ NWs have capacity of absorbing from visible to infrared light. Steady-state photoluminescence (PL) spectroscopy is then performed to examine the emission characteristics of the CsSnI_3_ NW thin films (Figs. 1f and S3c). Compared with the CsSnI_3_ NW film without BMIMCl, the peak intensity of the CsSnI_3_ NW film gradually increases with increasing the concentration of BMIMCl and reaches a maximum at the concentration of 8 mg mL^−1^. Then, the peak intensity gradually decreases after further increasing the concentration of BMIMCl. This can be explained that the moderate BMIMCl concentration can effectively passivate the defects of the CsSnI_3_ NWs and suppress the non-radiative recombination of the perovskites [23, 32]. Besides, the time-resolved PL indicates that the CsSnI_3_ NW film with 8 mg mL^−1^ BMIMCl shows longest carrier lifetime (Fig. S4d), which further confirms moderate BMIMCl concentration is beneficial for passivating the defects of the CsSnI_3_ NWs.

**Fig. 2:**
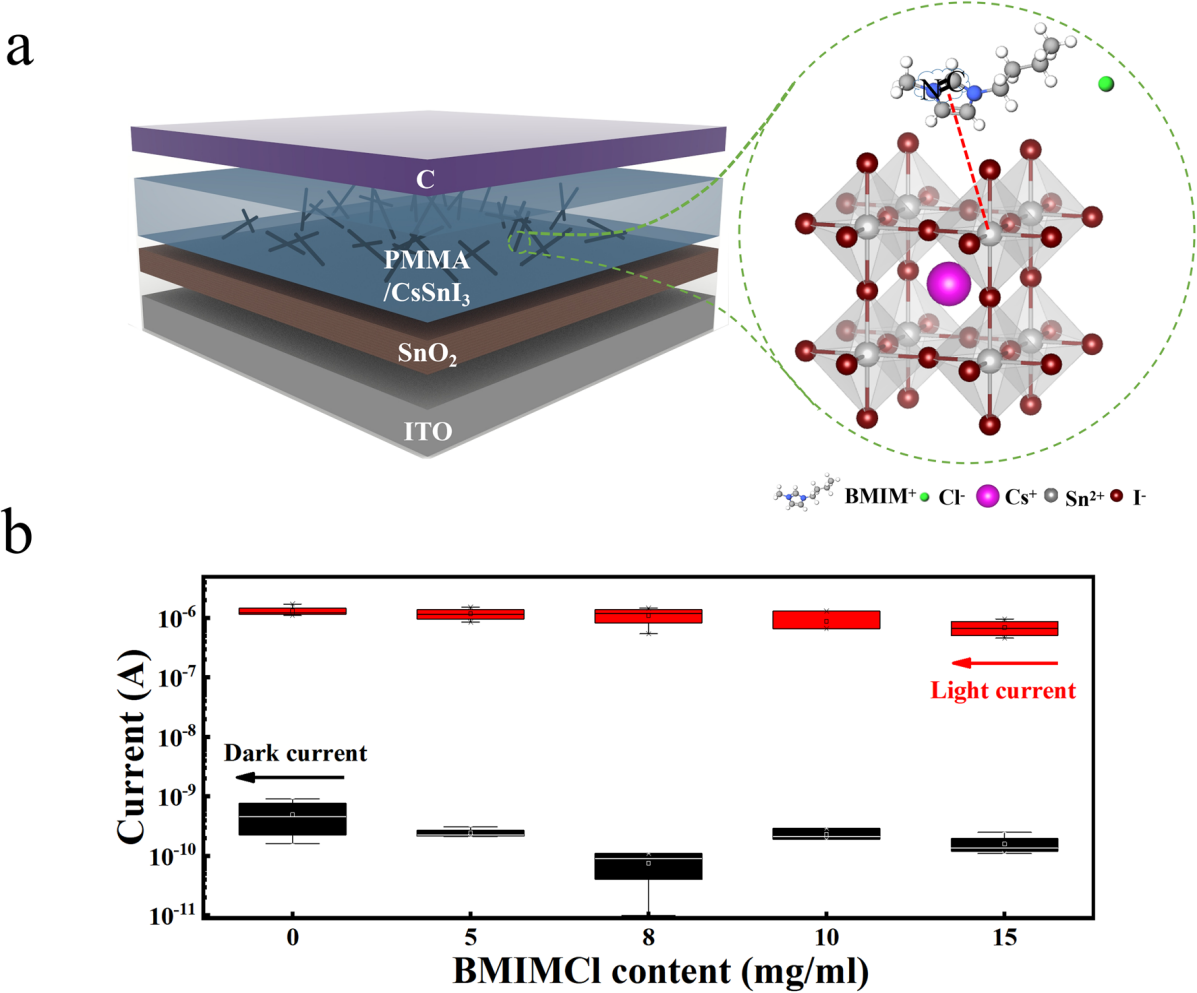
**a** Schematic structure of a PD based on the CsSnI_3_ with BMIMCl. **b** Device light/dark current statistic data under the addition of different levels of BMIMCl

Figure 2a shows the CsSnI_3_ NWPD with an ITO/SnO_2_/perovskite NWs/carbon structure, in which SnO_2_ is used as the electron transport layer, perovskite NWs are the photo-active layer, and carbon is as the top electrode also protects perovskites from corrosion by water and oxygen in air. As a high-performance PD, low dark current is essential, which has a positive impact on the linear dynamic range, signal-to-noise ratio and detectivity of the device. The relationship between the light and dark current of the PDs is shown in Fig. 2b, and 8 devices are used for the light/dark current statistic. The dark current gradually decreases as the BMIMCl concentration increases, and the dark current reaches a minimum until the concentration is at 8 mg mL^−1^. The drop in dark current is attributed to the passivation of the surface defects of perovskite, especially the Sn vacancies, which are the main culprit for the material to appear metallic [21]. However, too much ionic liquid will cause additional impurities, which will increase the dark current of the device. For the photocurrent, it shows a slightly downward trend. Considering the light and dark currents comprehensively, we believe that the NWPDs prepared at 8 mg mL^−1^ are best.

**Fig. 3:**
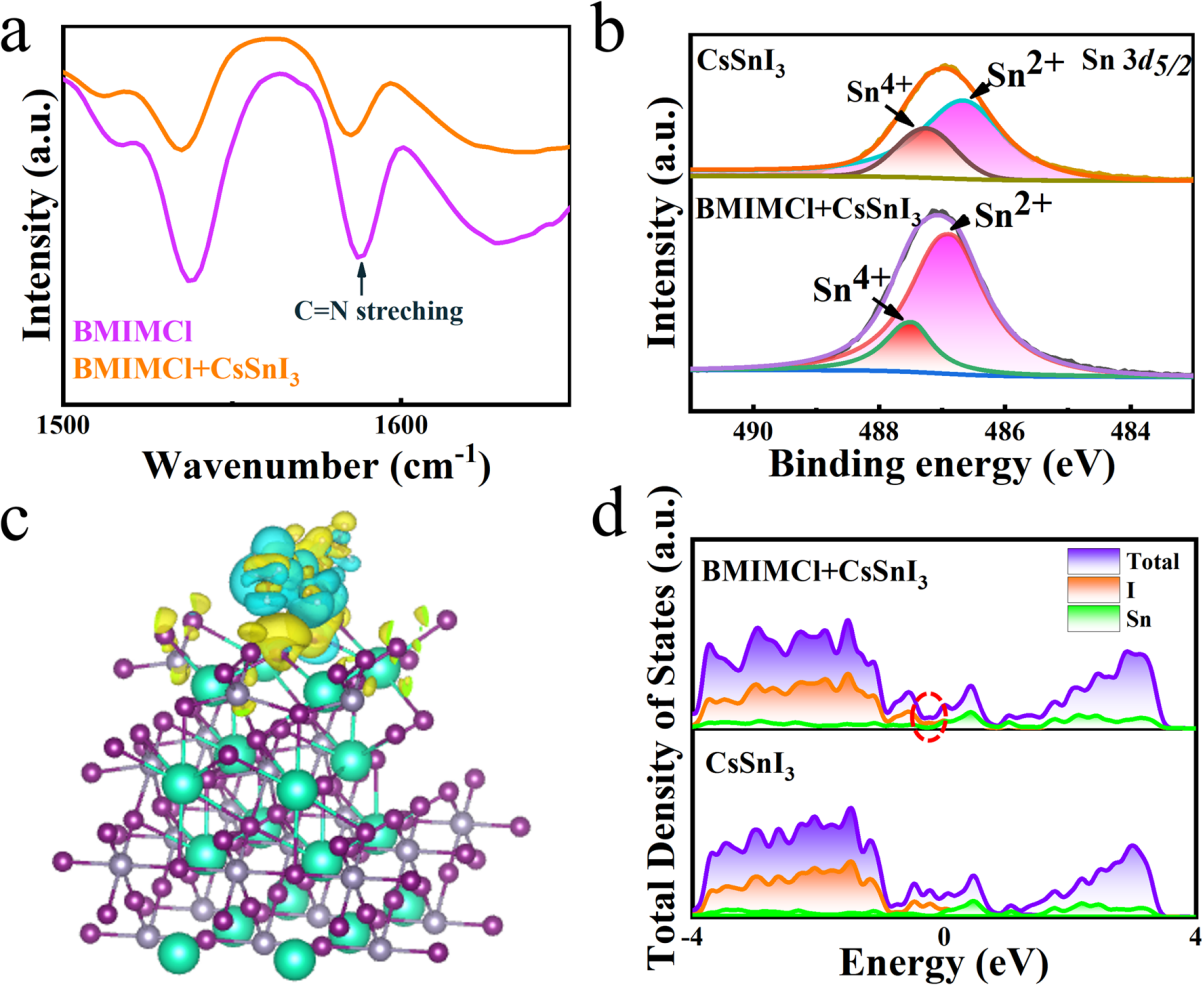
**a** FTIR spectra of the BMIMCl and CsSnI_3_ with BMIMCl at the wavenumber of 1500–1650 cm^−1^. **b** XPS spectra of Sn 3*d* of the CsSnI_3_ without and with BMIMCl. **c** Differential charge density of the BMIMCl on CsSnI_3_. **d** Total density of state of the perovskite and the BMIMCl passivated perovskite

To investigate the passivation interaction of the BMIMCl on Sn-related defects and verify our conjecture, Fourier transform infrared spectroscopy (FTIR) measurements are performed on BMIMCl and BMIMCl + CsSnI_3_. The peak of the pure BMIMCl sample is located at 1589 cm^−1^ (Fig. 3a), which corresponds to the N–C = N stretching vibration of the imidazole ring. For the BMIMCl + CsSnI_3_ sample, this peak is red-shifted to 1584 cm^−1^, indicating a strong interaction between N–C = N and CsSnI_3_. It is inferred that the N–C = N of BMIM^+^ has a strong passivating interaction with Sn-related defects such as Sn vacancies. The trap density of the Sn-based perovskites is strongly associated with the Sn^4+^ content, which is considered as the Sn vacancies and can be distinguished by X-ray photoelectron spectroscopy (XPS) measurement. As shown in Fig. 3b, compared with ordinary CsSnI_3_ samples, the proportion of Sn^4+^ in the BMIMCl + CsSnI_3_ sample is greatly reduced, and the proportion of Sn^2+^ rises, which indicates that the oxidation of Sn^2+^ to Sn^4+^ in the BMIMCl + CsSnI_3_ sample is significantly suppressed. In order to further verify our conjecture, the DFT calculations are used to study the effect of the introduction of BMIMCl on CsSnI_3_. Since the BMIMCl is coated on the surface of CsSnI_3_ perovskite, a model of BMIMCl on CsSnI_3_ is established, and the calculated differential charge density of BMIMCl + CsSnI_3_ (220) indicates the effective charge extraction of BMIMCl, as shown in Fig. 3c. Besides, the density of state (DOS) of CsSnI_3_ perovskites with or without BMIMCl is calculated, as shown in Fig. 3d. It can be clearly observed that compared with CsSnI_3_, the DOS of CsSnI_3_ after BMIMCl passivation is significantly reduced in electron defects near the Fermi level, indicating the reduction in charge carrier recombination in CsSnI_3_ after BMIMCl passivation and the promotion of electron transport in the perovskite.

Previous reports showed that the PMMA can fill the voids of the nanowires, passivate the surface defects of the perovskites [33] and can also generate Fowler–Nordheim tunneling to enhance device performance [34]. Based on these, we further adopt the PMMA to reduce the dark current and enhance the device performance. Through optimize the rotational speed, the appropriate thickness of the PMMA is obtained at 5000 rpm (Fig. S5a), and at this rotational speed, the device filled by the PMMA shows the lowest dark current and the biggest light current.

**Fig. 4:**
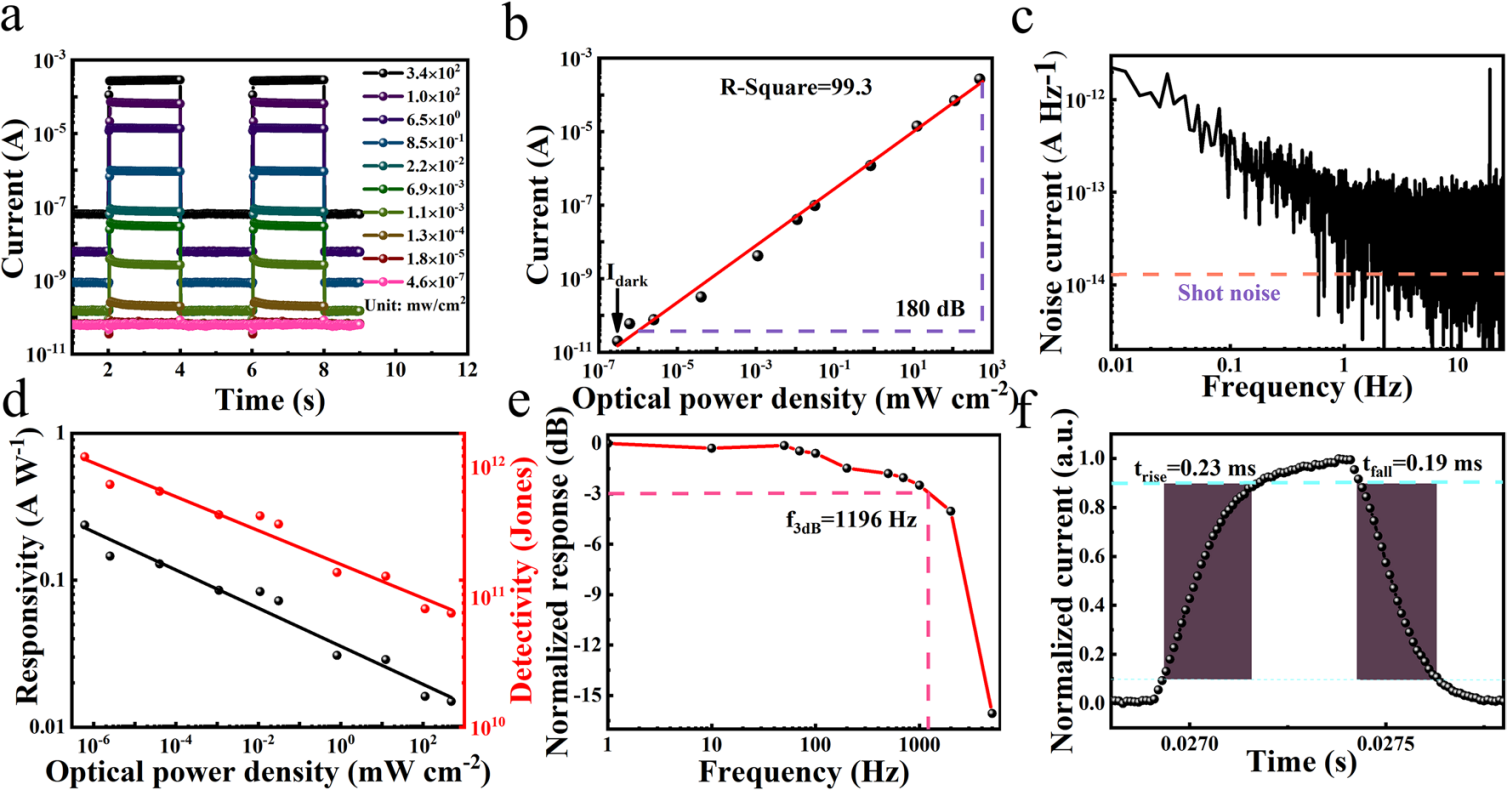
Device performances of the dual passivated PDs: **a** current–time curves at different light intensities; **b** LDR; **c** noise current curve; **d** responsivity and detectability *versus* light intensity curves; **e** f_-3 dB_ bandwidth; **f** response/recovery time. All curves are measured at 0 V

The detailed device performances are shown in Fig. 4. As shown in Fig. 4a, under the irradiation of 405 nm laser with different illumination intensities, our device exhibits good photoresponse characteristics to both strong and weak light. From this figure, there is a three-order-of-magnitude difference in dark current under different light intensities. We think the main reason of the three-order-of-magnitude increase in dark current is attributed to the perovskite CsSnI_3_, in which a few Sn vacancies still exist. When the device is illuminated by light, a large number of photo-generated carriers will be generated, and some of them may be trapped by these vacancies and remain. After the light is removed, these remaining photo-generated carriers will be slowly released and cause the rise of the dark current. When stronger light is applied, more photo-generated carriers are generated and more left, resulting in a three-order of magnitude difference in dark current under different light intensities.

The LDR of the PD is an important parameter for evaluating the performance of the PD, which can be calculated by the formula:

1 $${\text{LDR}} = 20{\text{ log}}\frac{{P_{{{\text{sat}}}} }}{{P_{{{\text{low}}}} }}$$

where *P*_sat_ and *P*_low_ are the strongest and weakest light intensities, respectively, when the incident light begins to deviate from the linear photocurrent. The calculated LDR is as high as 180 dB, indicating that our device can work stably in a wide range of the light intensity (Fig. 4b), and the ultra-low dark current of 2 × 10^–11^ A of the device is obtained. Besides, the noise of the device affects the sensitivity of the PD to light, and shot noise is an important noise source which affects the dark current. The device noise power spectrum (Fig. 4c) shows that the noise (*i*_n_) of the CsSnI_3_ NW PD is very low with the value of about 4 × 10^–13^ A Hz^−1^. Through the dark current, the shot noise of the device is further calculated, and it is close to the total noise, indicating that the main device noise is the shot noise. To further study the performance of the device, the responsivity *R* and the detectivity D* are measured to evaluate the sensitivity of the photoelectric response of the device to incident light, which are calculated by the formulas:

2 $$R = \frac{{I_{ph} }}{{{\text{AP}}_{{{\text{opt}}}} }}$$

3 $$D^{*} = \frac{{R\sqrt {{\text{AB}}} }}{{i_{n} }}$$

where *I*_ph_ is the photocurrent, *A* is the effective light irradiation area of the PD, *P*_opt_ is the light intensity, and *B* is the detection line width. As shown in Fig. 4d, both the *R* and the D* show a linear drop with increasing the light intensity. Under weak light, our PD shows high device performance with a *R* of 0.237 A W^−1^, and a D* of up to 1.18 × 10^12^ Jones, which are comparable to the reported high-performance Pb-based perovskite PDs and higher than those of the Pb-free perovskite PDs [35–44]; the detailed comparison is shown in Table S2. The achievement of the high performance is due to the dual passivation of perovskite defects through the BMIMCl and the PMMA, which greatly reduce the dark current, thereby reduce the device noise level and improve the detection capability of the device. Moreover, the broad light absorption range of the CsSnI_3_ enables the wide work region of our PD ranging from ultraviolet to infrared radiation, as shown in Fig. S5b. Furthermore, our device shows the f_-3 dB_ bandwidth of 1196 Hz (Fig. 4e) and the rise/fall time (τ_rise_/τ_fall_) of 0.23/0.19 ms (Fig. 4f).

**Fig. 5:**
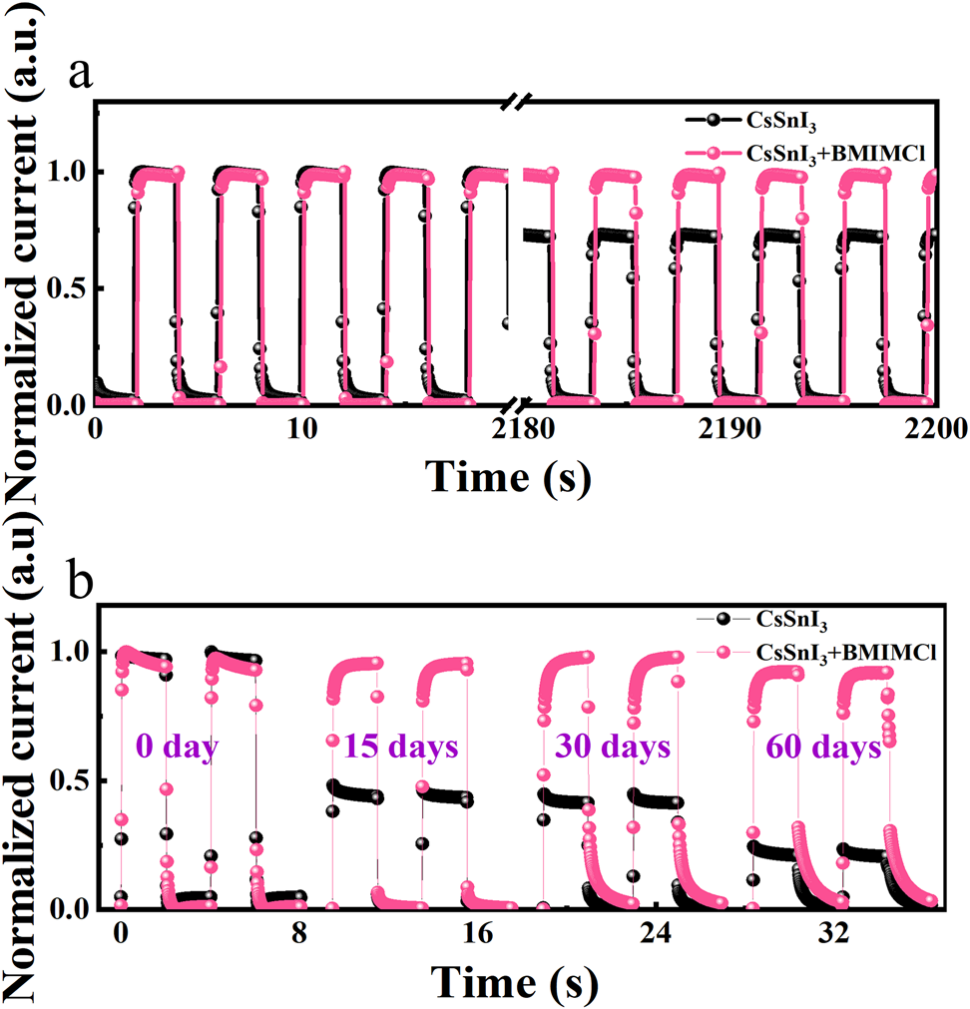
Stability of the PD under different conditions in air (25 °C, 50% humidity): **a** stability for long-time testing; **b** stability in long-term storage

Device stability is a key parameter of the PD, which is an important reference for commercialization. To confirm the improvement in device stability by the dual passivation strategies, we perform the stability of the unpackaged PDs under different conditions in air (25 °C, 50% humidity), as shown in Fig. 5. We find the performance of the passivated device shows no degradation when it is exposed to continuous light in air for more than 1200 s, but the light current of the original device indicates a significantly decline (Fig. 5a). We further place the devices in air for more than 60 days, and the performance of the original CsSnI_3_ PDs decreases to about 30% of the initial value. However, the device performance of the passivated devices remains 90% of the initial value after the same storage time. These results demonstrate that the introduction of the BMIMCl and PMMA not only enhances the optoelectronic performance of the device, but also improves the stability of the device.

## Conclusions

In conclusion, the BMIMCl was introduced in CsSnI_3_ NWs to passivate the Sn vacancies and reduce the dark current of the CsSnI_3_ NWPDs. Experimental analysis and theoretical calculation demonstrate that BMIM^+^ ions can effectively suppress the oxidation of Sn^2+^ to Sn^4+^ in the BMIMCl + CsSnI_3_ sample. For further reduce the dark current of the devices, the PMMA was applied, and finally, the dual passivated CsSnI_3_ NW PDs show ultra-high performance with an ultra-low dark current of 2 × 10^–11^ A, a responsivity of up to 0.237 A W^−1^, a high detectivity of 1.18 × 10^12^ Jones and a linear dynamic range of 180 dB. Furthermore, our unpackaged devices exhibit ultra-high stability in device performance after 60 days of storage in air (25 °C, 50% humidity), with the device performance remaining above 90%. This work provides a simple and effective method for the preparation of high-performance, highly stable Pb-free perovskite photoelectric devices.

## Supplementary Information

Below is the link to the electronic supplementary material.

Supplementary file1 (PDF 5711 kb)
