# Supplementary material for: Defect Passivation on Lead-Free CsSnI3 Perovskite Nanowires Enables High-Performance Photodetectors with Ultra-High Stability
Source: Nanomicro Lett. 2022 Nov 7;14:215. doi: 10.1007/s40820-022-00964-9 (PMC9640512; doi:10.1007/s40820-022-00964-9)
Supplement: Supplementary file 1 — Supplementary file1 (PDF 5711 kb) [file 40820_2022_964_MOESM1_ESM.pdf]

Supporting Information for

Defect Passivation on Lead-Free $CsSnI_3$ Perovskite Nanowires Enables High-Performance Photodetectors with Ultra-High Stability

Zheng Gao[1, 2, 3], Hai Zhou[1, *], Kailian Dong[2], Chen Wang[2], Jiayun Wei[3], Zhe Li[2], Jiashuai Li[2], Yongjie Liu[2], Jiang Zhao[3, *], Guojia Fang[2, *]

[1]International School of Microelectronics, Dongguan University of Technology, Dongguan, Guangdong 523808, P. R. China

[2]Key Lab of Artificial Micro- and Nano-Structures of Ministry of Education of China, School of Physics and Technology, Wuhan University, Wuhan 430072, P. R. China

[3]Faculty of Physics & Electronic Science, Hubei University, Wuhan 430062, P. R. China.

*Corresponding authors. E-mail: hizhou@dgut.edu.cn (Hai Zhou), zhaojiang@hubu.edu.cn (Jiang Zhao), gjfang@whu.edu.cn (Guojia Fang)

S1 Materials

DMF (Sigma-Aldrich, anhydrous, 99.8%); Isopropanol (Sigma-Aldrich, anhydrous, 99.9%); Chlorobenzene (CB, Sigma-Aldrich, 99.99%); Anhydrous methanol (Sigma-Aldrich, anhydrous, 98%); SnO_2 (Alfa Aesar, 15% in H_2O colloidal dispersion); Lead iodide (PbI_2, Advanced Election Technology Co., Ltd, China); PMMA (Aladdin); Tin(II) iodide (SnI_2, Advanced Election Technology Co., Ltd, China); Tin(II) fluoride (SnF_2, Aladdin); Cesium iodide (CsI, Sigma-Aldrich).

S2 Device Fabrication

A pre-etched ITO glass substrate was ultrasonically cleaned with detergent, DI water, ethanol, and IPA for 15 min, respectively. To prepare the SnO_2 precursor solution, the SnO_2 stock solution (1 mL) was diluted in deionized water (4 mL). The as-cleaned ITO substrate was treated with UV ozone at 100 °C for 10 min. A compact layer of SnO_2 was spin-coated on top of the ITO at 4000 rpm for 30 s. Then, it was heated at 150 °C for 30 min in air. After that, the samples were treated with UV ozone for 10 min. Subsequently, the samples were transferred into a N_2 filled glovebox with H_2O and O_2 concentrations of <0.1 ppm. A layer of PbI_2 film was fabricated by spin-coating PbI_2/BMIMCl (1 mol mL^{-1}/0, 5, 8, 10 and 15 mg mL^{-1}) in DMF at 3000 rpm for 30 s, followed by annealing at 70°C for 10 min. Then, the substrate was soaked in the prepared $CsI/SnI_2/SnF_2$ (5 /4 /0.4 mg ml^{-1}) solution in Anhydrous methanol for 2 h. After that, the substrates were placed in an isopropyl alcohol solution for 20 s, and then annealed at 180 °C for 10 min. Finally, a layer of PMMA was coated on the samples by spin-coating PMMA in CB (20 mg/ml) at 2000 /3000 /4000 /5000 /6000 /7000 rpm for 30 s, followed by annealing at 100 °C for 10 min. Then, a layer of carbon electrode was scraped on the samples and annealed at 120 °C for 15 min in air.

S3 Measurements

The SEM images of the $CsSnI_3$ nanowires were obtained from scanning electron microscopy (JSM7100F). The TEM images of the $CsSnI_3$ nanowires were performed by using the transmission electron microscopy (FEI/JEM). The ultraviolet-visible-near infrared (UV-VIS-NIR) absorption spectra of the $CsSnI_3$ nanowires were measured by using a UV-VIS-NIR spectrophotometer (MPC-3100SHIMADZU). The X-ray diffraction (XRD) patterns of the

$CsSnI_3$ nanowires were characterized by D8 FOCUS X-ray diffractometer with Cu Kα radiation at 40 kV and 40 mA. The X-ray photoelectron spectroscopy (XPS) of the $CsSnI_3$ nanowires were measured by using the Thermo Fisher Scientific Escalab 250Xi spectrometer with AI target Kα radiation source. The Fourier transform infrared (FTIR) spectra of the $CsSnI_3$ nanowires were recorded by using vacuum type fourier transform infrared spectrometer (BRUKER VERTEX 70V) in an attenuated total reflection mode.

S4 Device Characterizations

The current-voltage (*I-V*) and current-time (*I-t*) curves were measured by a Keysight B2912A Precision Sources/Measure Unit. A laser diode with wavelength of 405 nm was used as light source and its intensity was changed by using a series of neutral density filters, and its intensity was determined by a standard Si detector. All the devices measured in an ambient atmosphere.

S5 Calculations

All the calculations are performed in the framework of the density functional theory with the projector augmented plane-wave method, as implemented in the Vienna ab initio simulation package [S1]. The generalized gradient approximation proposed by Perdew, Burke, and Ernzerhof is selected for the exchange-correlation potential [S2]. The long-range van der Waals interaction is described by the DFT-D3 approach [S3]. The cut-off energy for plane wave is set to 400 eV. The energy criterion is set to 10^{-5} eV in iterative solution of the Kohn-Sham equation. The Brillouin zone integration is performed at the Gamma point for structural optimization, and a 3×3×1 k-mesh grid is used for electronic structure calculations. All the structures are relaxed until the residual forces on the atoms have declined to less than 0.05 eV $Å^{-1}$.

S6 Supplementary Figures and Tables

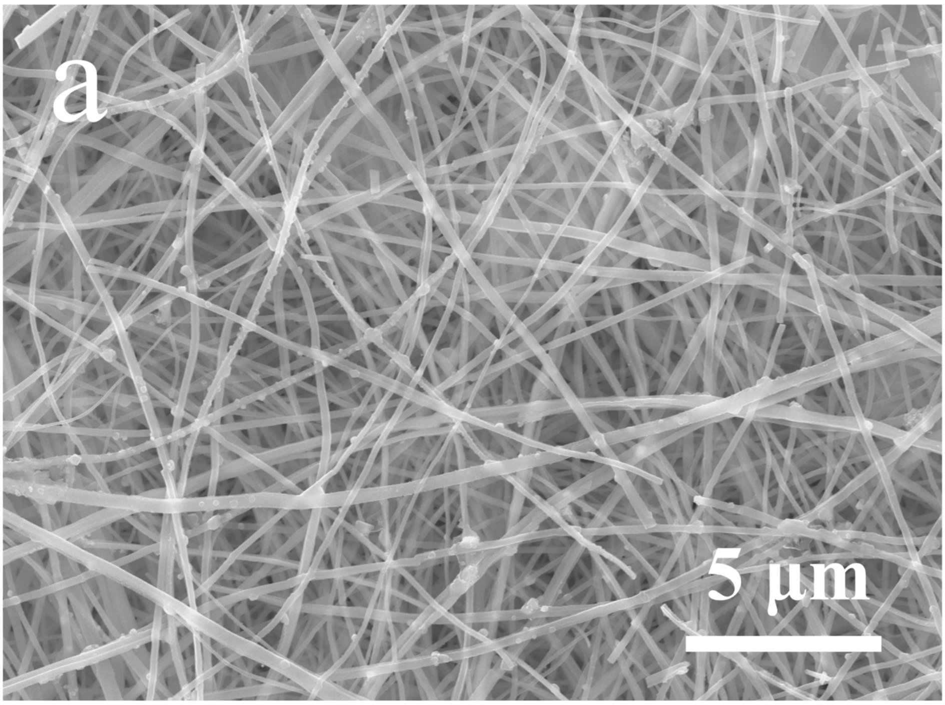

Fig. S1 Surface SEM images of the γ-$CsSnI_3$ NWs

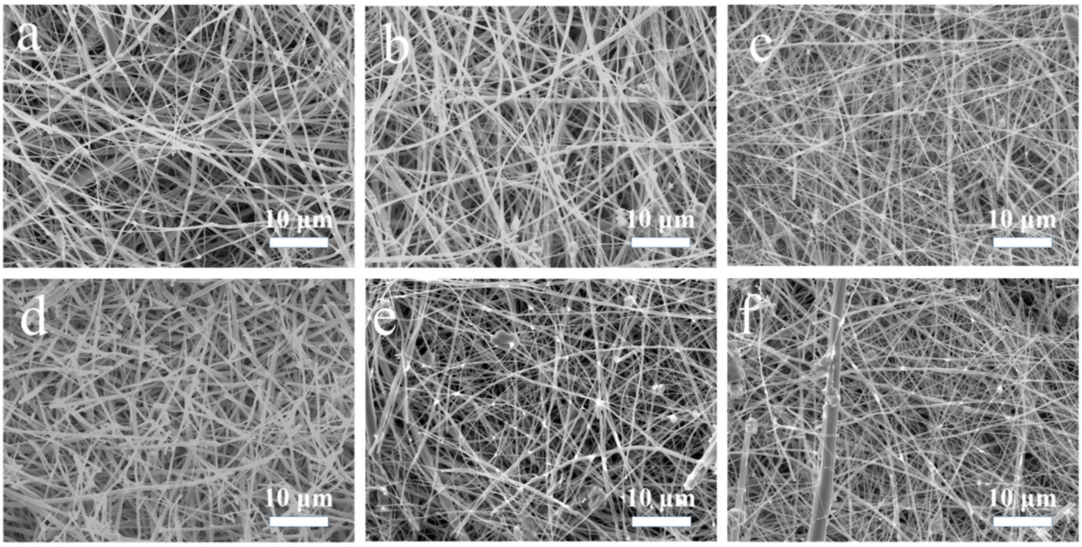

Fig. S2 Surface SEM images of the $CsSnI_3$ NWs with different soaking times: (**a**) 2 h; (**b**) 4 h; (**c**) 8 h; (**d**) 16 h; (**e**) 24 h; (**f**) 48 h

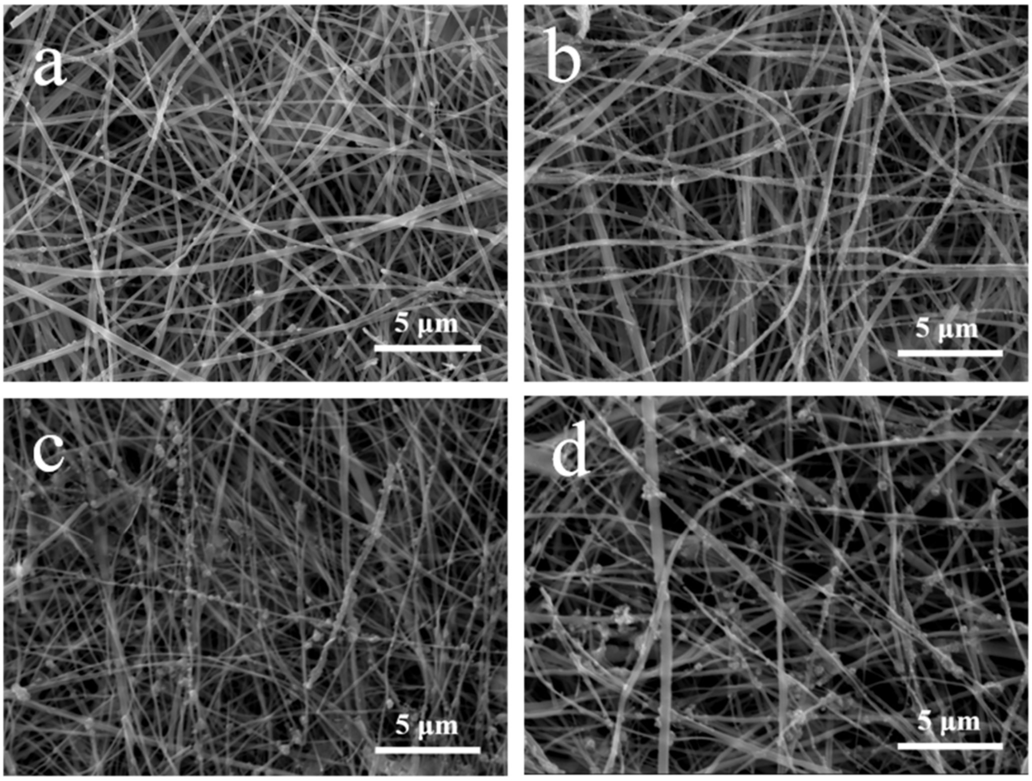

Fig. S3 Surface SEM images of $CsSnI_3$ NWs with different levels of BMIMCl: (**a**) 0 mg mL^{-1}; (**b**) 5 mg mL^{-1}; (**c**) 10 mg mL^{-1}; (**d**) 15 mg mL^{-1}

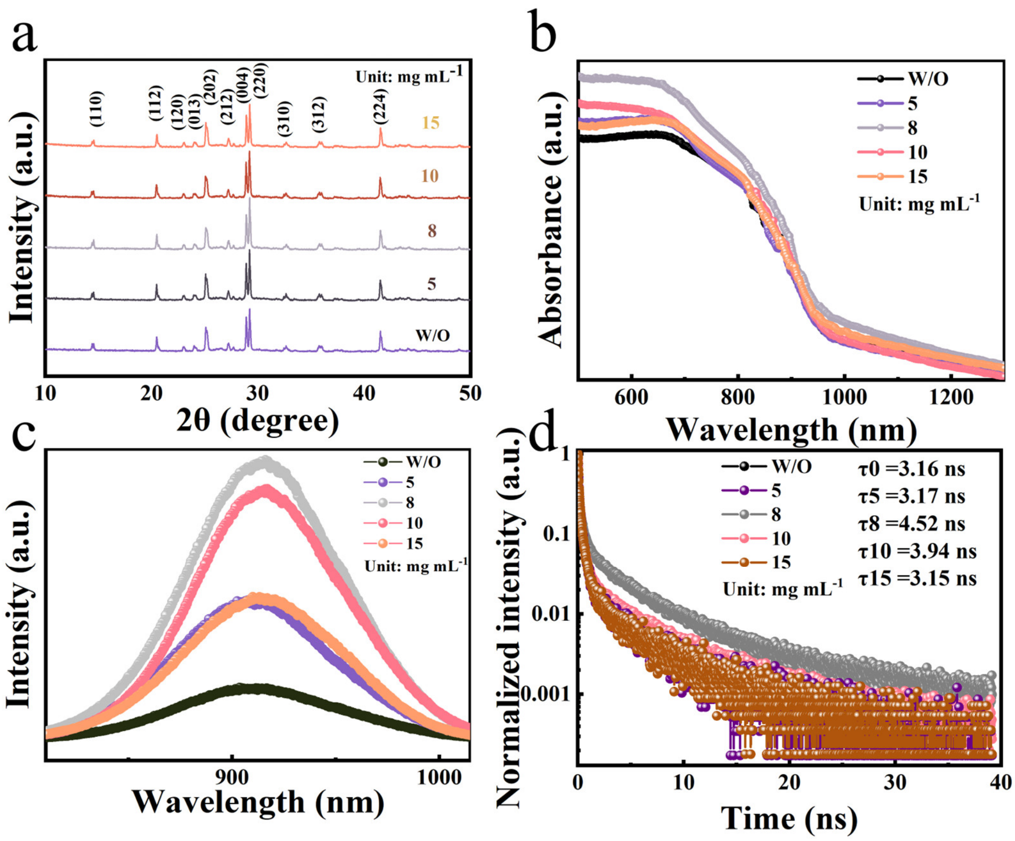

Fig. S4 (**a**) XRD, (**b**) optical absorption, (**c**) steady-state PL and (**d**) TRPL spectra of $CsSnI_3$ NWs with different levels of BMIMCl

Through exponential fitting, the carrier lifetime τ is calculated by the equation $\tau = \frac{A_1 t_1^2 + A_2 t_2^2}{A_1 t_1 + A_2 t_2}$, where A_1, A_2, t_1, t_2 are obtained by double exponential fitting to the data. The carrier lifetime τ of the devices is 3.16 (W/O), 3.17 (5 mg mL^{-1}), 4.52 (8 mg mL^{-1}), 3.94 (10 mg mL^{-1}), and 3.15 (15 mg mL^{-1}) ns, respectively.

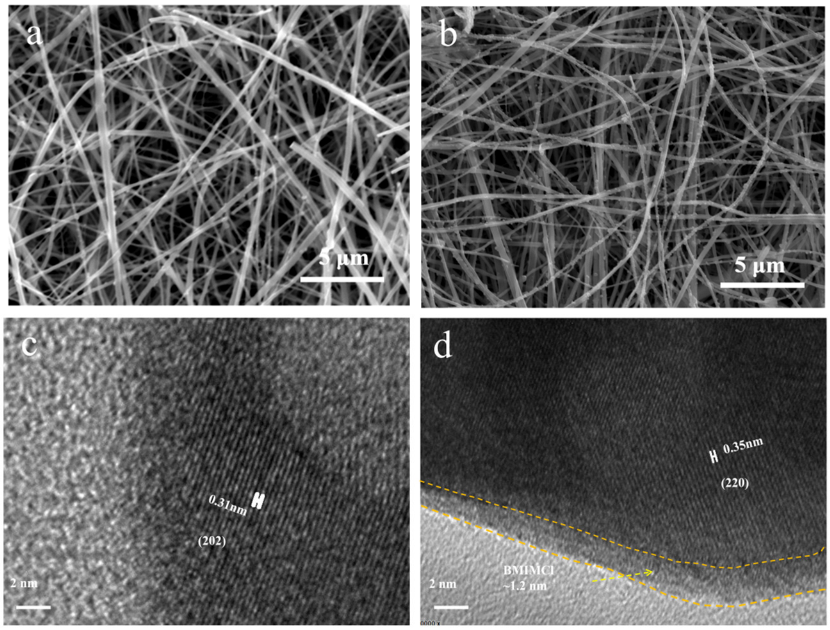

Fig. S5 (**a**) Surface SEM of $CsSnI_3$ NWs. (**b**) Surface SEM of BMIMCl+ $CsSnI_3$ NWs. (**c**) HRTEM of $CsSnI_3$ NWs. (**d**) HRTEM of BMIMCl+ $CsSnI_3$ NWs

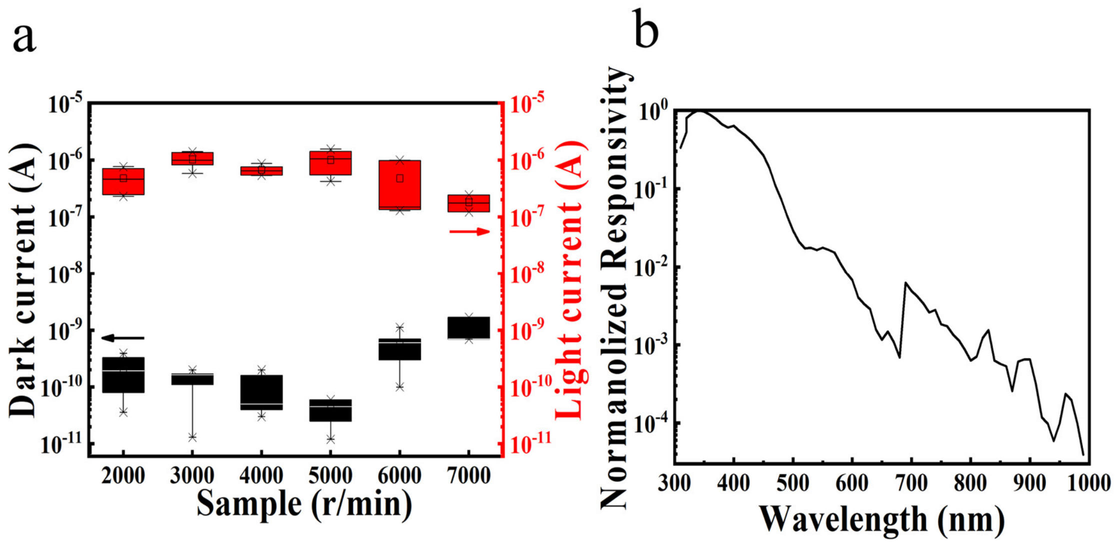

Fig. S6 (**a**) Device light/dark current statistic data under the different rotational speeds of spin-coating PMMA. (**b**) The spectral responsivity of the PD based on BMIMCl+ $CsSnI_3$+PMMA

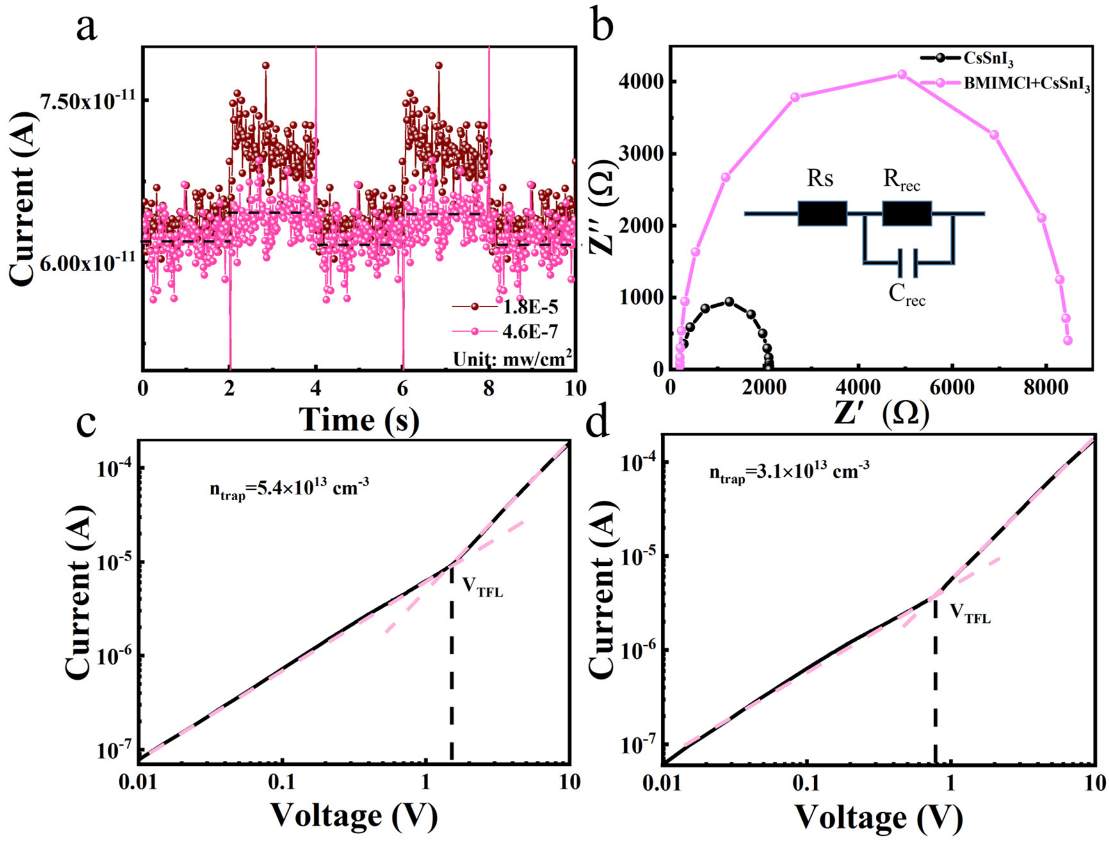

Fig. S7 (**a**) current-time curves under weak light. (**b**) Nyquist plots of PDs based on $CsSnI_3$ and BMIMCl+$CsSnI_3$ measured under dark conditions, the inset is the equivalent circuit used for the fit. (**c**) The dark J-V curves of photoconductive devices fabricated with $CsSnI_3$ and BMIMCl+$CsSnI_3$, indicating the lower defect density of states of the samples by defect passivation

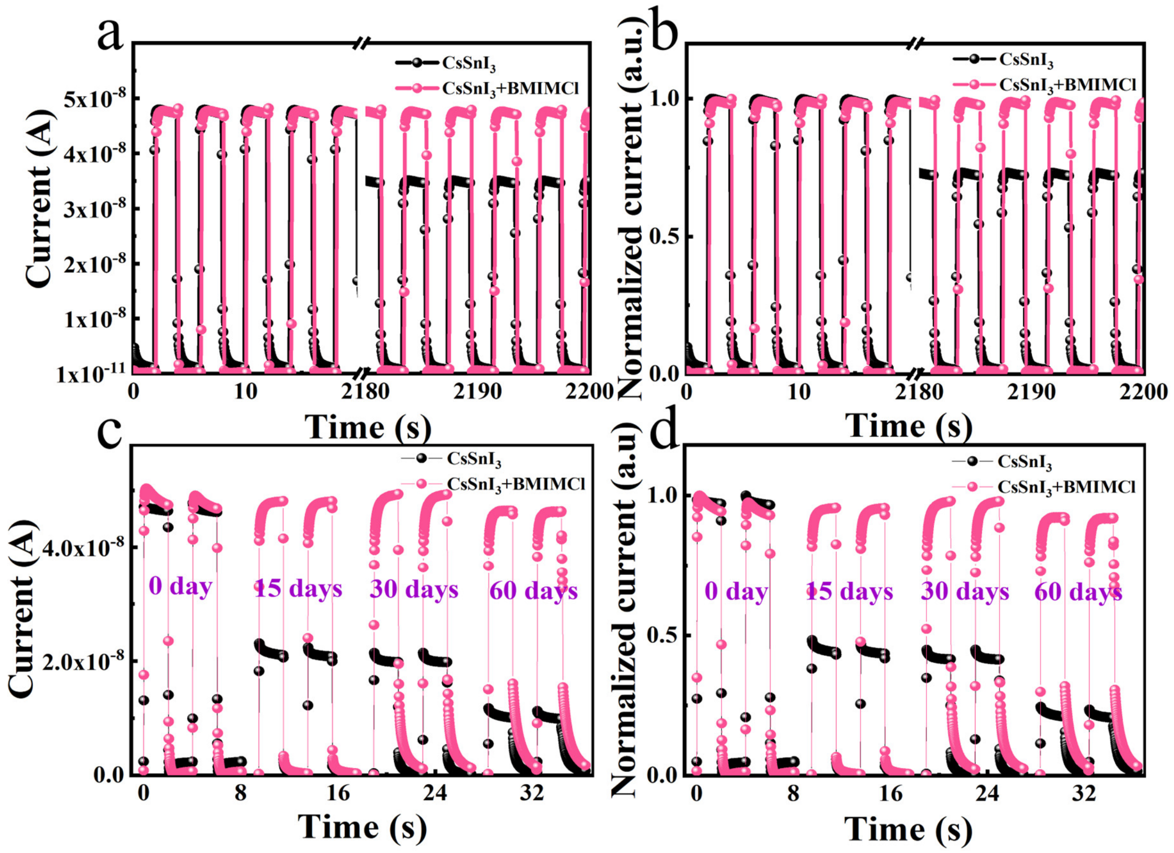

Fig. S8 Stability of the PD under different conditions in air (25°C, 50% humidity): (**a**) the current stability for long-time testing; (**b**) the normalized current stability for long-time testing; (**c**) the current stability in long-term storage; (**d**) the normalized current stability in long-term storage. All curves were obtained by using a laser irradiation of 405 nm with the optical power density of 7 mW/cm^2

Table S1 Elemental content of Sn and Pb in $CsSnI_3$ NWs in EDS at different soaking times

Elemental content	Soaking Time					
	2 h	4 h	8 h	16 h	24 h	48 h
Sn (%)	98.9	98.9	98.3	98.6	99.8	98.5
Pb (%)	1.1	1.1	1.7	1.4	0.2	1.5

Table S2 Comparison of the main parameters of the $CsSnI_3$ NW PDs with other Pb-based and Pb-free perovskite PDs in the literature

Perovskite	R (A W^{-1})	D* (Jones)	LDR (dB)	Refs.
$CsPbI_xBr_{3-x}$	0.28	9.70×10^{12}	200	[S4]
$MAPbI_3$	0.064	1.27×10^{12}		[S5]
$CsPbBr_3$	0.18	6.10×10^{10}		[S6]
$CsPbCl_3$	0.38	3.30×10^{11}		[S7]
$PEA_2MA_4Pb_5I_{16}$	0.25	1.40×10^{12}	120	[S8]
$CsPbI_2Br$	0.43	2.20×10^{11}	116	[S9]
$MASnI_3$	0.47	8.80×10^{10}		[S10]
$Cs_3Bi_2I_9$	~0.02	3.90×10^{11}		[S11]
$Cs_3Sb_2I_9$	0.04	1.26×10^{11}		[S12]
$CsCu_2I_3$	0.27	6.38×10^{8}		[S13]
$CsSnI_3$	0.237	1.18×10^{12}	180	This work

Supplementary References

[S1] G. Kresse, D. Joubert, From ultrasoft pseudopotentials to the projector augmented-wave method. Phys. Rev. B **59**, 1758 (1999). https://doi.org/10.1103/PhysRevB.59.1758

[S2] J.P. Perdew, K. Burke, M. Ernzerhof, Generalized gradient approximation made simple. Phys. Rev. Lett. **77**, 3865 (1996). https://doi.org/10.1103/PhysRevLett.77.3865

[S3] S. Grimme, J. Antony, S. Ehrlich, H. Krieg, A consistent and accurate ab initio parametrization of density functional dispersion correction (DFT-D) for the 94 elements H-Pu. J. Chem. Phys. **132**, 154104 (2010). https://doi.org/doi/10.1063/1.3382344

[S4] C. Bao, J. Yang, S. Bai, W. Xu, Z. Yan et al., High performance and stable all-inorganic metal halide perovskite-based photodetectors for optical communication applications. Adv. Mater. **30**(38), 1803422 (2018). https://doi.org/10.1002/adma.201803422

[S5] J. Wang, S. Xiao, W. Qian, K. Zhang, J. Yu et al., Self-driven perovskite narrowband photodetectors with tunable spectral responses. Adv. Mater. **33**(3), 2005557 (2021). https://doi.org/10.1002/adma.202005557

[S6] X. Li, D. Yu, F. Cao, Y. Gu, Y. Wei, Y. Wu, J. Song, H. Zeng. Healing all-inorganic perovskite films via recyclable dissolution–recyrstallization for compact and smooth carrier channels of optoelectronic devices with high stability. Adv. Funct. Mater. **26**(32), 5903-5912 (2016). https://doi.org/10.1002/adfm.201601571

[S7] X. Wu, J. Sun, H. Shao, Y. Zhai, L. Li et al., Self-powered UV photodetectors based on $CsPbCl_3$ nanowires enabled by the synergistic effect of acetate and lanthanide ion passivation. Chem. Eng. J. **426**, 131310 (2021). https://doi.org/10.1016/j.cej.2021.131310

[S8] F. Zhu, G. Lian, B. Yu, T. Zhang, L. Zhang et al., Pressure-enhanced vertical orientation and compositional control of ruddlesden–popper perovskites for efficient and stable solar cells and self-powered photodetectors. ACS Appl. Mater. Interfaces **14**(1), 1526-1536(2021). https://doi.org/10.1021/acsami.1c18522

[S9] J. Li, G. Zhang, Z. Zhang, J. Li, Z. Uddin et al., Defect passivation via additive engineering to improve photodetection performance in $CsPbI_2Br$ perovskite photodetectors. ACS Appl. Mater. Interfaces **13**(47), 56358-56365 (2021). https://doi.org/10.1021/acsami.1c19323

[S10] A. Waleed, M.M. Tavakoli, L. Gu, Z. Wang, D. Zhang et al., Lead-free perovskite nanowire array photodetectors with drastically improved stability in nanoengineering templates. Nano Lett. **17**(1), 523-530 (2017). https://doi.org/10.1021/acs.nanolett.6b04587

[S11] Z. Li, X. Liu, C. Zuo, W. Yang, X. Fang, Supersaturation-controlled growth of monolithically integrated lead-free halide perovskite single-crystalline thin film for high-sensitivity photodetectors. Adv. Mater. **33**(41), 2103010 (2021). https://doi.org/10.1002/adma.202103010

[S12] S.K. Shil, F. Wang, Z. Lai, Y. Meng, Y. Wang et al., Crystalline all-inorganic lead-free $Cs_3Sb_2I_9$ perovskite microplates with ultra-fast photoconductive response and robust thermal stability. Nano Res. **14**(11), 4116-4124 (2021). https://doi.org/10.1007/s12274-021-3351-x

[S13] X. Xu, C. Fan, Z. Qi, S. Jiang, Q. Xiao et al., $CsCu_2I_3$ nanoribbons on various substrates for UV photodetectors. ACS Appl. Nano Mater. **4**(9), 9625-9634 (2021). https://doi.org/10.1021/acsanm.1c02041
